# Reassessing Impacts of Extended Daily Exposure to Low Level Solar UV Radiation

**DOI:** 10.1038/s41598-018-32056-3

**Published:** 2018-09-14

**Authors:** Richard L. McKenzie, Robyn M. Lucas

**Affiliations:** 1 National Institute of Water & Atmospheric Research (NIWA), Lauder, New Zealand; 2 National Centre for Epidemiology and Population Health, Australian National University, Canberra, Australia

## Abstract

Currently, health agencies recommend that no sun-protection is required when the UV Index (UVI) is less than 3. We use high-quality data from spectroradiometers and model calculations to demonstrate that this simplification is seriously flawed, particularly for mid-latitude conditions. For days when the peak UVI is below the threshold for advising protection, the daily dose of sun-burning UV available frequently far exceeds the threshold for damage to fair skin. This may have important health consequences, as populations at mid latitudes include a significant proportion with fair skin that is susceptible to damage.

## Introduction

Information on the risks of sun exposure is usually communicated to the public in terms of the UV Index (UVI), which is a measure of “sun-burning” (i.e., erythemally-weighted) UV irradiance (UV_Ery_) incident on a horizontal surface^1^. When UV_Ery_ is measured in units of Wm^−2^, the UVI is then simply 40x UV_Ery_^2^. Thus, the UVI is a measure of the rate of deposition of energy, or in other words, the power. Currently, WHO INTERSUN recommend that ‘no protection is necessary’ when the UVI is less than 3^2^. Some agencies go on to define a “UV Alert” period as the period of the day during which the UVI is three or more, again implying that no protection is required when the UVI is less than 3.

Such messages, that are based only on UVI^1^ (i.e. irradiance), rather than UVI *and* duration, are flawed, and potentially contribute to increased health risks. The problem arises because skin damage is a function of the dose of UV (i.e., the energy, measured in Joules) rather than the dose rate (i.e., the power, measured in Watts (or Joules per second)). The ‘UVI equals 3′ threshold corresponds to the dose rate for which damage to fair skin would take approximately 1 hour. Taking a UVI of less than 3 as a threshold for no sun protection presumes that nobody will stay in the sunlight for more than an hour: a presumption that is quite arbitrary, and clearly incorrect. For example, golf is one of the world’s most popular sports, and it typically takes about 4 hours to complete a round.

The dose of erythemally weighted UV radiation can be expressed in terms of the number of Standard Erythemal Doses (SEDs), where 1 SED = 100 Jm^−2^. For fair skinned individuals, the dose required to elicit visible damage, as indicated by the first signs of skin reddening 24 hours after exposure, is typically around 2.0 to 2.5 SED^1^. In other words, for fair skinned individuals, the dose required to achieve a minimum sunburn (the Minimum Erythemal Dose (MED)) is about 2.5 SED. Furthermore, it has been demonstrated that the damaging effect of exposure to a larger UVI value for a short time, is similar to that from a low UVI value for a commensurately longer time^3^. For example, the skin damaging effect of 2.5 SED from UVI = 15 for about 11 minutes is similar to that for UVI = 1.5 for 110 minutes. That is: reciprocity holds between dose rate (UVI) and time. The study quoted shows that reciprocity in erythema applies for exposure times from 1 second to 1 hour. Little work has been done for lower dose rates (e.g., UVI < 3), where it could be argued that repair mechanisms may be able cope adequately. However, a recent study showed an approximately linear increase in epidermal DNA damage (as measured by cyclobutane pyrimidine dimers (CPDs) in skin biopsies) for doses in the range 0.2 to 1.0 MED^4^.

## Method

We investigated the relationship between the peak daily UVI and the cumulative daily dose in SEDs using data from multiple measurements each day, taken with UV spectroradiometer systems that meet the demanding standards required by the Network for the Detection of Atmospheric Composition Change (NDACC)^5^. Each spectrum takes approximately 7 minutes to acquire, and represents the average of a forward-scan and a reverse scan over the wavelength range 285–450 nm. Spectra are taken at 15-minute intervals over the 2.5-hour period centred on local solar noon, and at 5-degree steps in solar zenith angle outside that range. Typically, this provides 25 to 30 spectra per day. These data are available on the NDACC web site, at http://www.ndsc.ncep.noaa.gov/UVSpect_web/. Corresponding calculations for clear-sky conditions were also undertaken using the TUV radiative transfer model^6^.

## Results

**Figure 1:**
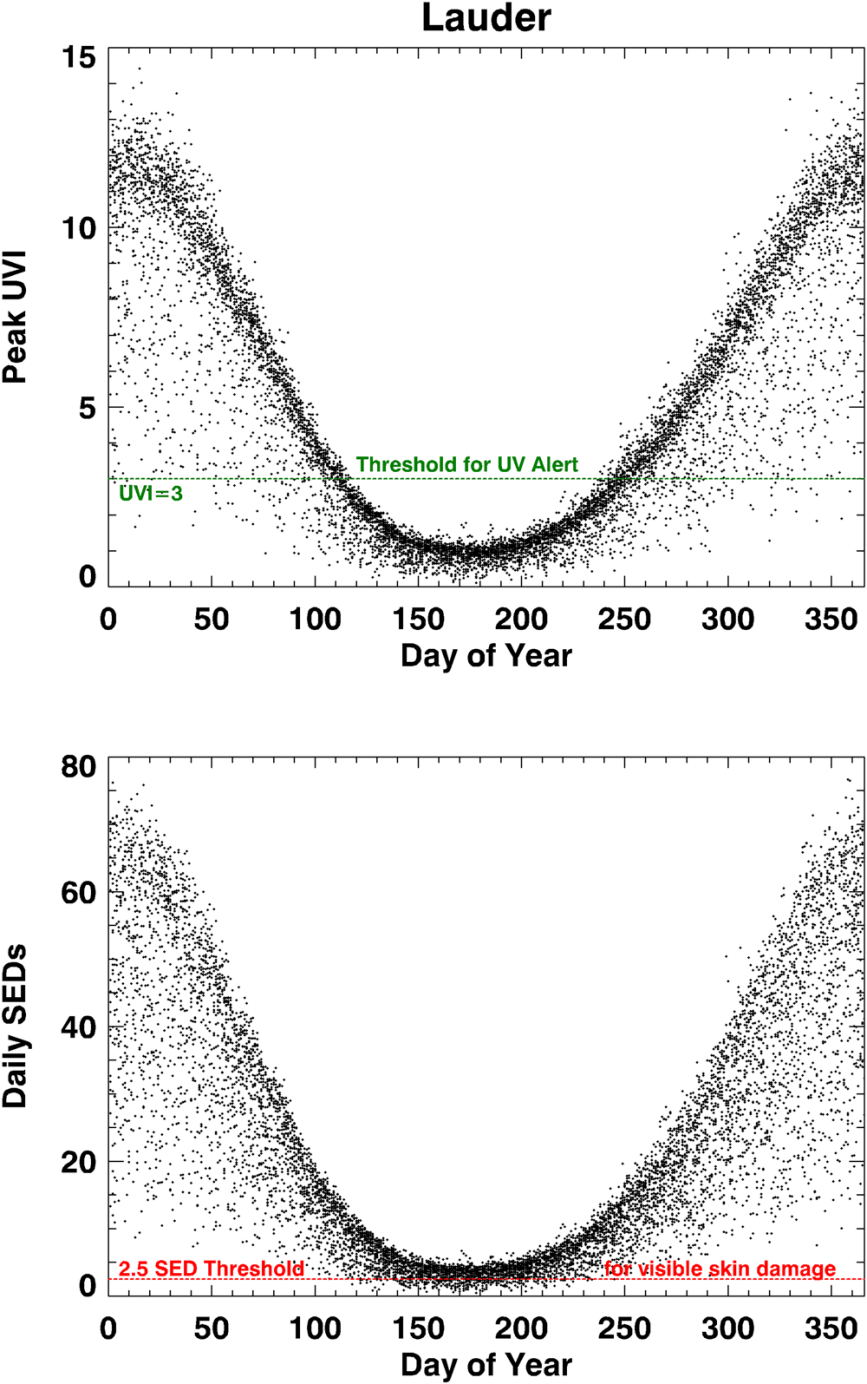
Annual variability of peak UVI and daily dose in SEDs measured at Lauder New Zealand.

The variability of peak UVI and daily dose in SEDs over the course of a year at a pristine mid-latitude site, Lauder New Zealand (45°S, 170°E, alt: 370 m) is shown in Fig. 1. The data are from approximately 9000 days for which there were sufficient data to allow valid estimates of daily doses over the 25-year period dating from the 1990s to the present. Over that period, no significant trends in UVI, e.g. as a result of stratospheric ozone depletion or changes in air quality, have been detected at this site^7^.

In summer at this site, the peak UVI measured during the ~5-minute scan period can exceed 14, and the peak daily dose can exceed 75 SED. Thus, over the course of a summer’s day there is sufficient sun-burning UV radiation to cause 30 erythemal doses (i.e., 30 MEDs) for fair skin (and this in turn means that if a sunscreen is to provide “all day” protection, it must, by definition, have an SPF greater than 30). Even on the shortest day in mid-winter at this site, when the peak UVI for clear skies is only ~1, the daily dose is still close to ~4 SED. And over the winter months of June, July, and August, the mean of the peak daily UVI is 1.25, and the mean daily dose is 4.85 SED. Thus, on the average winter day at this site there are nearly 2 MEDs available.

**Figure 2:**
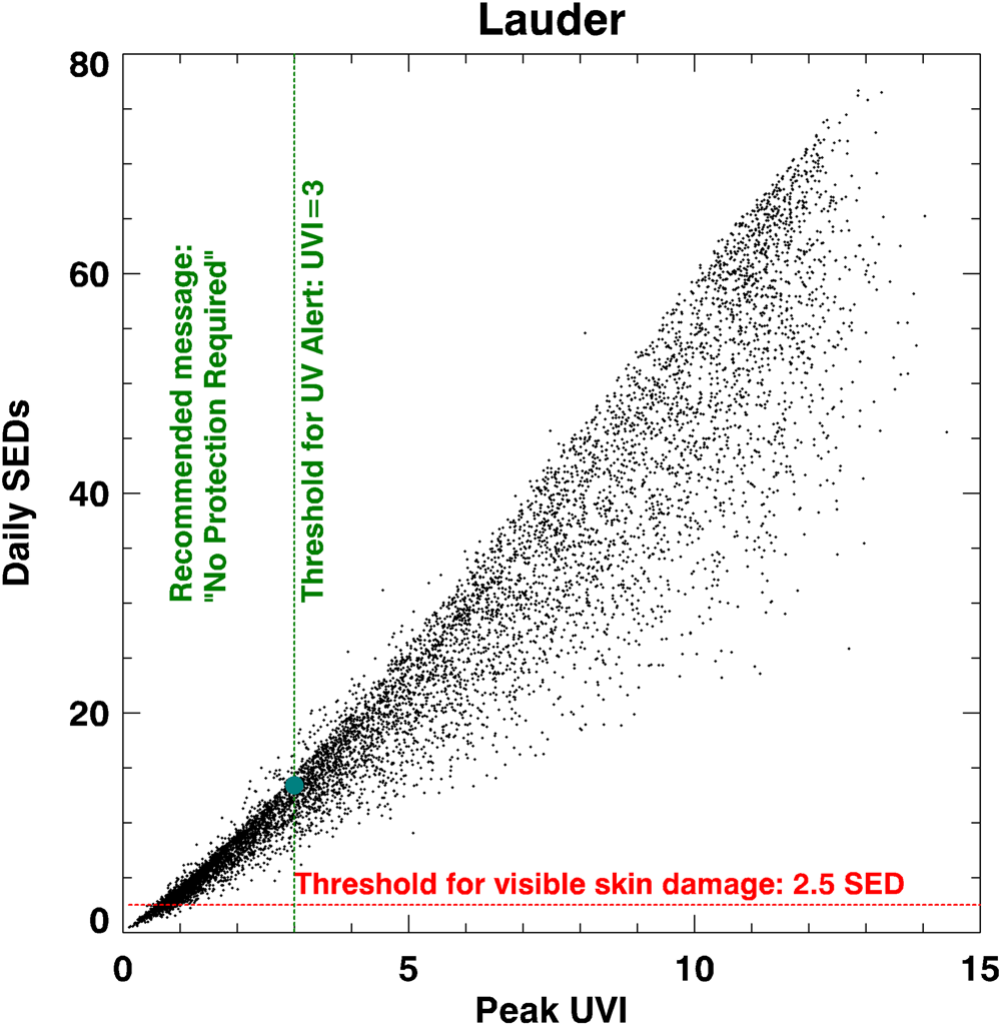
Daily dose of erythemally-weighted UV as a function of the peak UVI measured at Lauder New Zealand. The blue point shows the mean SED for UVI = 3, calculated for clear skies at Lauder.

Figure 2 shows the daily dose of erythemally-weighted irradiance (in SEDs) as a function of the peak UVI that day. The plot shows that there are very few days for which the daily dose is less than 2.5 SED (=1 MED for fair skin): approximately only one day per year. Of note here, is the large proportion of days for which the peak UVI is less than 3, yet the daily dose is greater than 2.5 SED. In fact, the figure shows that the damage threshold for fair skin is exceeded on virtually all days, including those for which the peak UVI is in the range 0.5 to 3.0. There are frequent days for which the UVI is less than 3, yet the daily dose exceeds 10 SEDs, and on a few occasions, it reaches 15 SEDs (Table 1).

**Table 1:** Approximate number of days per year, based on multi-year data from several sites, for UVI ≥ 3 and UVI < 3, with the latter subdivided according to daily MED values, where 1 MED is taken as 2.5 SED.

Site	Lat	UVI ≥ 3	UVI < 3			
			All SED	Daily Dose <1 MED (no. erythema possible)	Daily Dose >1 MED (erythema possible)	Daily Dose >2 MED (erythema possible In <2.5 hours)
Lauder, NZ	−45	219	146	13	133	72
Melbourne, Aus.	−38	239	126	2	124	101
Alice Springs, Aus.	−20	360	5	0	4	4
Mauna Loa, HI	20	365	0	0	0	0
Boulder, CO	40	258	107	5	102	86

Similar spectroradiometer systems are deployed at other sites, covering a wide range of latitudes (e.g., Melbourne (38°S) and Alice Springs (20°S), in Australia; and Boulder CO (40°N) and Mauna Loa Observatory Hawaii (20°N), in USA). All sites exhibit similar patterns (see Fig. S1, in the Supplementary Data), with daily doses at the mid-latitude sites frequently exceeding 10 SED when the peak UVI remains less than 3. At the tropical sites (Mauna Loa and Alice Springs) there are only a small number of days for which the UVI is less than 3, but the daily doses on those days still usually exceed the visible skin damage threshold by at least a factor of 3.

Table 1 shows that at Lauder, the peak UVI is less than 3 for about 146 days each year, but the daily dose is less than 2.5 SED on only 13 of those days. On the other 133 days, erythema can occur with sufficient duration of exposure, so the advice that ‘no protection is required’ is misleading. Indeed, on 72 of those days, more than two MEDs are possible. We note that in mid-winter at Lauder (45°S), there are only 8.5 daylight hours, and that 50% of the daily erythemal dose falls within the 2.5-hour period centred on local solar noon. Consequently, if the daily dose exceeds 5 SED, then an erythemal dose is possible within 2.5 hours for exposure periods centred on solar noon. When the maximum daily dose exceeds 10 SED, visible skin damage can occur in little more than 1 hour in a fair-skinned person.

The statistics for a range of sites show that this flaw in advice, whereby sunburn is easily achievable when the UVI is <3 and no protection is advised, is mainly a mid-latitude problem. The issue is less important in the winter at higher latitudes, such as Northern Europe (e.g., Germany, UK)^8,9^, where the peak UVI is less than 1, and, from the relationships demonstrated here (Fig. S1), daily UV doses will generally be less than 2.5 SED for much of the winter. However, similar issues will arise in the shoulder seasons when the peak values approach UVI = 3.

Although spectroradiometers provide the most accurate UVI measurements available^10^, their disadvantage is that continuous measurements are not possible; and with typically only 20–30 spectra per day, uncertainties can result from the daily variation from spectroradiometer data being under-sampled in time. We estimate that the uncertainties in daily dose could be as large as 10% for a single day, which would translate to uncertainties of ±7 days in the last 2 columns of Table 1.

To investigate further whether this is an issue, we used the TUV radiative transfer model^6^, to calculate UVI at 1 minute intervals and then integrated these results over time to calculate the UV daily dose, in SEDs, as a function of the peak daily UVI for clear sky conditions. Calculations were performed for a ~12-year period at Lauder, representing approximately 4400 days.

These calculations show excellent agreement with the measurements. Calculated values closely follow the upper envelope of data in Fig. 2 that correspond to clear-sky days. For a given peak daily UVI, the daily doses can vary by ±5–10 percent, due to differences in day-length and ozone amount. To avoid clutter, the calculated value is shown only for the UVI = 3 threshold value, for which the mean calculated daily dose is 13.4 SED, with a range from 12.5 to 14.3 SED. The calculations confirm that for clear-skies at Lauder, the daily dose can exceed 14 SED when the peak UVI is less than 3 and can exceed 11 SED when the peak UVI is less than 2.5. In the former case, mild erythema could result from exposure periods of approximately 1 hour near solar noon.

## Discussion

We have shown that information provided to the public on sun protection is flawed. The findings are particularly relevant for mid-latitude conditions. For days when the peak UVI is below the threshold for advising protection, the daily dose of sun-burning UV available frequently far exceeds the threshold for visible damage to fair skin. Furthermore, the damaging UV received on exposed surfaces, such as the face or neck, may be more pronounced than implied from these measurements of UVI during winter, because when the sun is lower in the sky, the UV incident on such surfaces can be significantly greater than on a horizontal surface. The head and neck are the body sites for which skin damage from sunlight is most pronounced.

This incorrect advice to the public may have important public health consequences. The frequency of days with peak UVI less than 3, but with daily doses exceeding the threshold for damage is largest at the three mid-latitude sites considered. For each of these sites, the dose on days in which the peak UVI is less than 3 represents approximately 10% of the total annual dose. In contrast, at the two low-latitude sites, the contribution from days with UVI < 3 is less than 1% of the total. In the limit, at the highest northern latitudes, the peak UVI is always less than 3^11^, and 100% of the total annual dose is received on days with the peak UVI less than 3 (though in this case, the daily total dose will usually be below the threshold for damage).

Populations at mid latitudes include a significant proportion with fair skin that is susceptible to UV damage. Because ‘no protection’ is advised, precautions against sunburn are unlikely to be taken, even for long-duration outdoor activities. If a sunscreen of SPF 30 was correctly applied on days with UVI > 3, but not applied on days with UVI < 3, over the course of a year, the cumulative dose to the skin on the low UVI days would exceed that from the high UVI days by a factor of 3. Thus, the messaging unnecessarily increases risks to health, such as the development of skin cancer.

Advice that ‘no protection is required’ for UVI < 3 should be removed from public messaging. Furthermore, because skin damage depends on both the UVI and the time of exposure, that is the dose rather than the dose rate, behavioural messages should be further tailored to reflect the time needed, rather than having a simple binary message such as ‘protection required’ (for UVI ≥ 3) or ‘no protection required’ (for UVI < 3). The specific ‘no protection’ advice should be reserved only for days where the cumulative daily total UV dose is less than the damage threshold (e.g., 2.5 SED for skin type II).

## Electronic supplementary material

Supplementary Information
