# Supplementary material for: Reassessing Impacts of Extended Daily Exposure to Low Level Solar UV Radiation
Source: Sci Rep. 2018 Sep 14;8:13805. doi: 10.1038/s41598-018-32056-3 (PMC6138670; doi:10.1038/s41598-018-32056-3)
Supplement: Supplementary file 1 — Supplementary Information [file 41598_2018_32056_MOESM1_ESM.pdf]

Reassessing Impacts of Extended Daily Exposure to Low Level Solar UV Radiation

Richard L. McKenzie, Robyn M. Lucas

Supplementary Information

The relationship between peak UVI and daily dose in SEDs is shown below for other sites midlatitude and tropical sites below (Figure S1). Note that the measured and calculated thresholds for these sites are similar to that for Lauder (Figure 2).

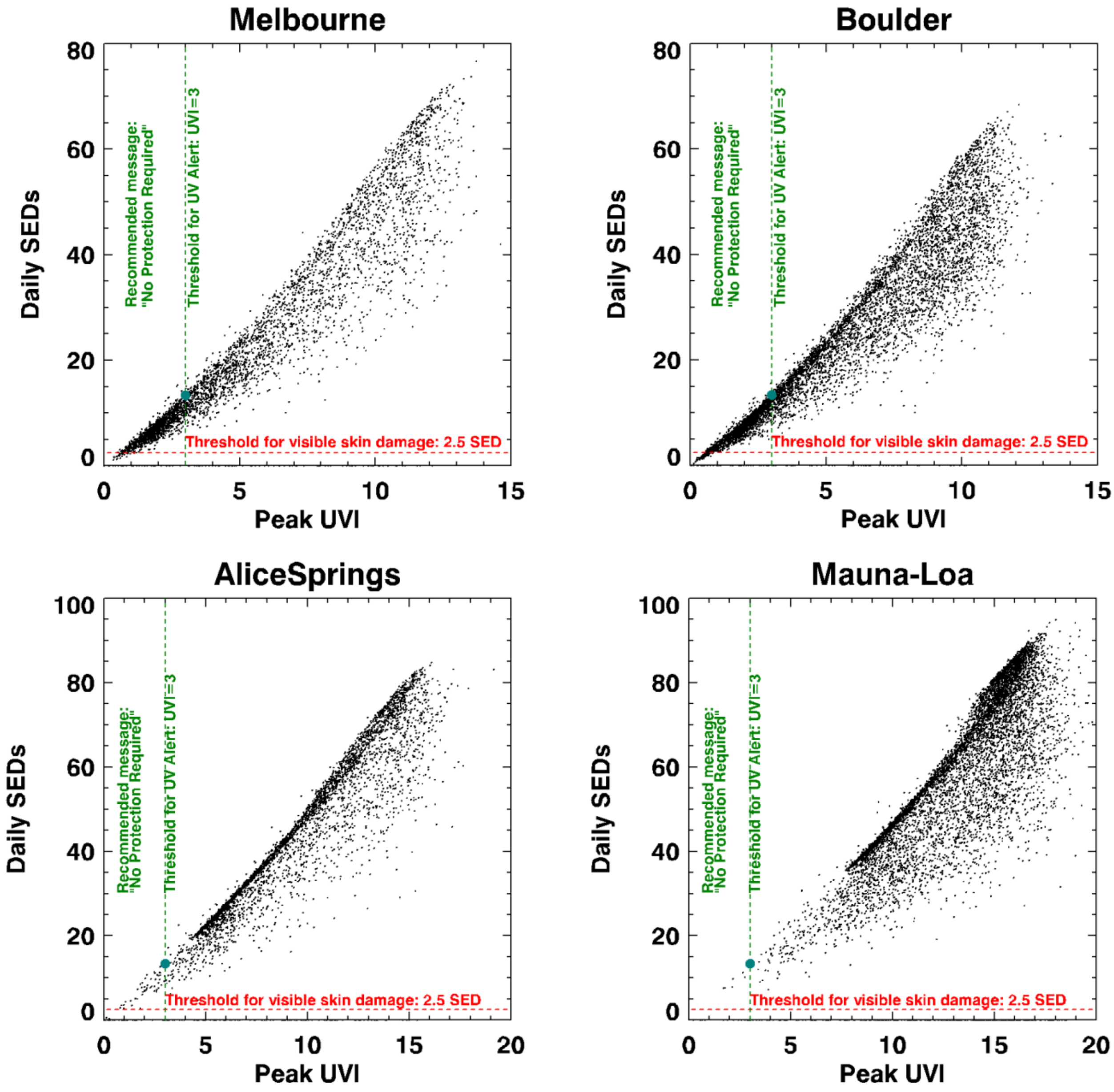

Figure S1. As for Fig. 2, but for two sites in Australia (left panels), and two in USA (right panels). The upper panels are mid-latitude sites, and the lower panels are tropical sites. Note the expanded scale ranges, and reduced frequency of UVI values less than 3 at the tropical sites. The blue point in each panel shows the mean SED for UVI=3, calculated for clear skies at Lauder.
